# Dehydration affects cerebral blood flow but not its metabolic rate for oxygen during maximal exercise in trained humans

**DOI:** 10.1113/jphysiol.2014.272104

**Published:** 2014-05-16

**Authors:** Steven J Trangmar, Scott T Chiesa, Christopher G Stock, Kameljit K Kalsi, Niels H Secher, José González-Alonso

**Affiliations:** 1 Centre for Sports Medicine and Human Performance, Brunel UniversityLondon, UK; 2 Department of Anaesthesia, Rigshospitalet, University of CopenhagenCopenhagen, Denmark

## Abstract

Intense exercise is associated with a reduction in cerebral blood flow (CBF), but regulation of CBF during strenuous exercise in the heat with dehydration is unclear. We assessed internal (ICA) and common carotid artery (CCA) haemodynamics (indicative of CBF and extra-cranial blood flow), middle cerebral artery velocity (MCA *V*_mean_), arterial–venous differences and blood temperature in 10 trained males during incremental cycling to exhaustion in the heat (35°C) in control, dehydrated and rehydrated states. Dehydration reduced body mass (75.8 ± 3 *vs*. 78.2 ± 3 kg), increased internal temperature (38.3 ± 0.1 *vs*. 36.8 ± 0.1°C), impaired exercise capacity (269 ± 11 *vs*. 336 ± 14 W), and lowered ICA and MCA *V*_mean_ by 12–23% without compromising CCA blood flow. During euhydrated incremental exercise on a separate day, however, exercise capacity and ICA, MCA *V*_mean_ and CCA dynamics were preserved. The fast decline in cerebral perfusion with dehydration was accompanied by increased O_2_ extraction (*P* < 0.05), resulting in a maintained cerebral metabolic rate for oxygen (CMRO_2_). In all conditions, reductions in ICA and MCA *V*_mean_ were associated with declining cerebral vascular conductance, increasing jugular venous noradrenaline, and falling arterial carbon dioxide tension (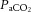) (*R*^2^ ≥ 0.41, *P* ≤ 0.01) whereas CCA flow and conductance were related to elevated blood temperature. In conclusion, dehydration accelerated the decline in CBF by decreasing 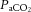 and enhancing vasoconstrictor activity. However, the circulatory strain on the human brain during maximal exercise does not compromise CMRO_2_ because of compensatory increases in O_2_ extraction.

**Key points**

- Dehydration accrued during exercise in the heat challenges systemic and locomotor muscle blood flow, but its impact on cerebral blood flow (CBF) and metabolism remains unknown.
- This study assessed whether dehydration compromises CBF and the cerebral metabolic rate for oxygen (CMRO_2_) during incremental exercise to exhaustion in trained males.
- Dehydration induced an early reduction in CBF during progressive exercise, but increased O_2_ extraction secured CMRO_2_.
- In all hydration conditions declining CBF at high exercise intensities was correlated to decreasing arterial carbon dioxide tension and increasing jugular venous plasma noradrenaline.
- These results suggest that dehydration impairs CBF at high exercise intensities, but this circulatory strain on the human brain does not compromise CMRO_2_.

## Introduction

Heat stress, with or without dehydration, compromises blood flow to active muscles and skin during strenuous exercise as the systemic circulation becomes compromised (González-Alonso & Calbet, 2003; González-Alonso *et al*. 2008; Crandall & González-Alonso, 2010). Intense exercise in the heat is also associated with a marked decline in middle cerebral artery blood velocity (MCA *V*_mean_), suggesting attenuated cerebral perfusion (Nybo & Nielsen, 2001a,b; González-Alonso *et al*. 2004). Changes in MCA *V*_mean_, however, may not reflect alterations in cerebral blood flow (CBF) as the vessel cross-sectional area remains unknown (Madsen *et al*. 1993; Jørgensen, 1995; Wilson *et al*. 2011; Willie *et al*. 2012). Additionally, dehydration intensifies the effect of heat stress on active muscle blood flow and increases the rate of heat storage in part by attenuating skin perfusion (Sawka *et al*. 1985; González-Alonso *et al*. 1995, 1998; Montain *et al*. 1998; Cheuvront *et al*. 2010). It remains unknown, however, whether dehydration affects CBF during maximal incremental exercise in the heat and, if so, how that is established.

On the transition from rest to moderate exercise, regional and global CBF increase to support neuronal activity (Ide & Secher, 2000; Secher *et al*. 2008; Ogoh & Ainslie, 2009a). However, CBF reaches a plateau or declines to baseline values prior to the attainment of maximal work rate (Madsen *et al*. 1993; Moraine *et al*. 1993; Hellström *et al*. 1996; Ide & Secher, 2000; Sato *et al*. 2011). During intense exercise, restricted cerebral perfusion could challenge the cerebral metabolic rate for oxygen (CMRO_2_) (Nybo & Rasmussen, 2007; Rasmussen *et al*. 2010) and in part explain the orthostatic intolerance and reduced motor output with heat stress (Van Lieshout *et al*. 2003; Wilson *et al*. 2006; Brothers *et al*. 2009a; Nelson *et al*. 2011; Ross *et al*. 2012; Bain *et al*. 2013). Alternatively, reduced CBF can be compensated by increased oxygen extraction such that CMRO_2_ is maintained or increased (Nybo *et al*. 2002; González-Alonso *et al*. 2004). Whether the CMRO_2_ remains adequate during strenuous exercise in the heat with concomitant dehydration is yet unknown.

Understanding the mechanisms restricting CBF in intensely exercising humans is important for devising strategies that could ameliorate or delay its potential deleterious effects. During exercise, attenuation of CBF is in part due to cerebral vessel vasoconstriction, concomitantly with an increased systemic and regional cerebral sympathetic activity, increasing body temperature, and reduced arterial carbon dioxide tension (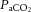) (Wilson *et al*. 2002; Querido & Sheel, 2007; Fan *et al*. 2008; Secher *et al*. 2008; Seifert & Secher, 2011). The cerebral vasculature is highly sensitive to changes in 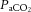, with elevations resulting in vasodilatation and reductions leading to vasoconstriction (Kety & Schmidt, 1948; Ogoh & Ainslie, 2009b; Willie *et al*. 2012). At rest, these responses are of importance for maintenance of a stable pH across the brain and reflect the sensitivity of the brainstem to acute changes in CO_2_. However, 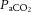 only accounts for ∼7% of the CO_2_ transported from the cerebral tissue whereas the majority of CO_2_ is bound to haemoglobin (23%) or buffered as bicarbonate (70%). If local tissue pH balance is important for regulation of CBF, blood CO_2_ content (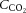) could account for the alterations in cerebrovascular tone. It is also evident that changes in CO_2_ are not associated with changes in conduit artery and extra-cranial (i.e. common (CCA) and external carotid artery (ECA)) tone and perfusion, as blood flow in these vessels increases progressively with exercise intensity (Hellström *et al*. 1996; Sato *et al*. 2011). Extra-cranial blood flow is likely to be controlled by thermoregulatory, rather than pH regulatory mechanisms (Fan *et al*. 2008; Sato *et al*. 2011, 2012; Bain *et al*. 2013; Ogoh *et al*. 2013); yet direct evidence for a relationship between flow and blood temperature is lacking. While evidence indicates differences in blood flow responses to exercise at the vascular beds perfusing the head, the impact of dehydration on graded exercise in the heat and the potential roles of 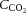, 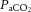 and blood temperature in these responses have not been investigated.

The purpose of this study was to investigate cerebral and extra-cranial blood flow and CMRO_2_ during incremental exercise to exhaustion in the heat, with and without dehydration, and to provide insights into the vascular mechanisms underpinning these responses. CBF was measured using Doppler ultrasonography, and arterial to internal jugular venous differences for oxygen, CO_2_ and noradrenaline were measured for assessment of the exchange of these substances across the brain. We hypothesised that dehydration would accelerate the attainment of maximal CCA blood flow but also accentuate the reduction in CBF during exercise in association with the lowering of 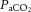 and 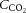 and the increase in sympathetic activity, and yet increased O_2_ extraction would maintain or enhance CMRO_2_.

## Methods

### Ethical approval

Fully informed, written consent was obtained from the participants prior to the study. All procedures were approved by the Brunel University Research Ethics Committee (RE07-11) and conformed to the guidelines of the *Declaration of Helsinki*.

### Participants

Ten healthy experienced cyclists (mean ± SD; age 29 ± 5 years, stature 183 ± 5 cm, mass 78 ± 9 kg and 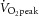 59 ± 6 ml kg^−1^ min^−1^) participated in the study. All participants were non-smokers and free from cardio-respiratory, metabolic and neurological disease. Participants arrived at the laboratory postprandial with a normal hydration status and were required to have abstained from strenuous exercise and alcohol intake for 24 h and caffeine consumption for 12 h.

### Experimental design

The participants visited the laboratory for three preliminary sessions followed by two experimental sessions, each separated by at least 1 week. On the first session the participants were introduced to the experimental set-up and familiarised with the methodology. Investigation of the extra-cranial arteries and MCA *V*_mean_ Doppler spectra determined the reliability of images and identified the temporal ultrasound window and the position for the best signal-to-noise ratio. Participants performed incremental exercise on a semi-recumbent cycle ergometer (Lode Angio, Groningen, the Netherlands) with a backrest inclination of 45 deg, to establish the maximal work rate (WR_max_), maximal heart rate, and 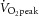. The initial work rate was 20 W for 3 min, followed by step increments of 60 W every 3 min until the limit of tolerance. Pedal cadence was maintained between 70 and 90 r.p.m. and the test was terminated when it dropped below 60 r.p.m., for more than 3 s, despite strong verbal encouragement to continue. On the second and third visits, participants cycled in an environmental chamber set at 35°C (relative humidity (RH) 50%) in the semi-recumbent position for 2 h at 55% WR_max_ with heart rate and intestinal temperature recorded. No fluid consumption was permitted during exercise and body mass was recorded before and immediately post exercise.

**Figure 1:**
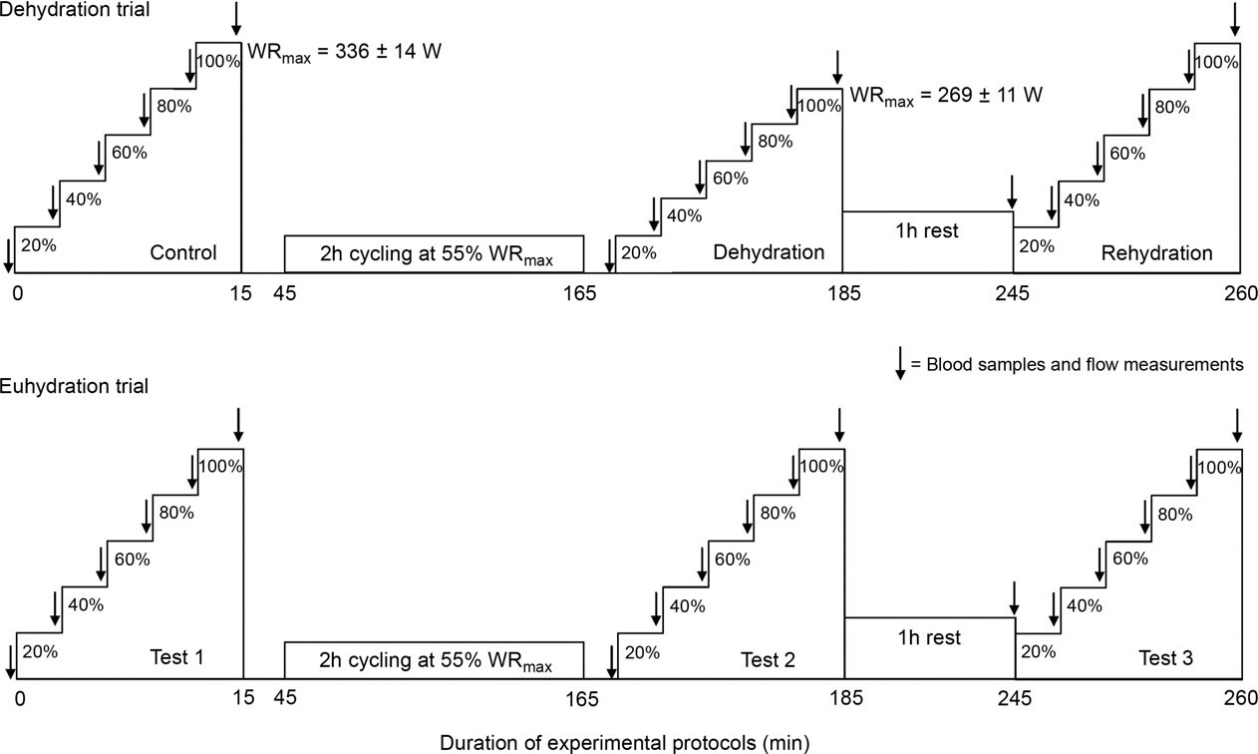
Experimental protocol Schematic representation of the experimental protocols. Participants completed 2 trials (i.e. dehydration and euhydration trials) separated by at least 1 week. Each trial consisted of 3 incremental cycle ergometer exercise tests until volitional exhaustion. The incremental exercise consisted of five, 3 min stages at 20, 40, 60, 80 and 100% of WR_max_. In the dehydration trial, WR_max_ was approximately 20% lower when participants were dehydrated compared to when they were euhydrated or rehydrated (269 ± 11 *vs*. 336 ± 14 W). In the euhydration trial, however, WR_max_ was the same in the 3 incremental exercise tests.

**Figure 2:**
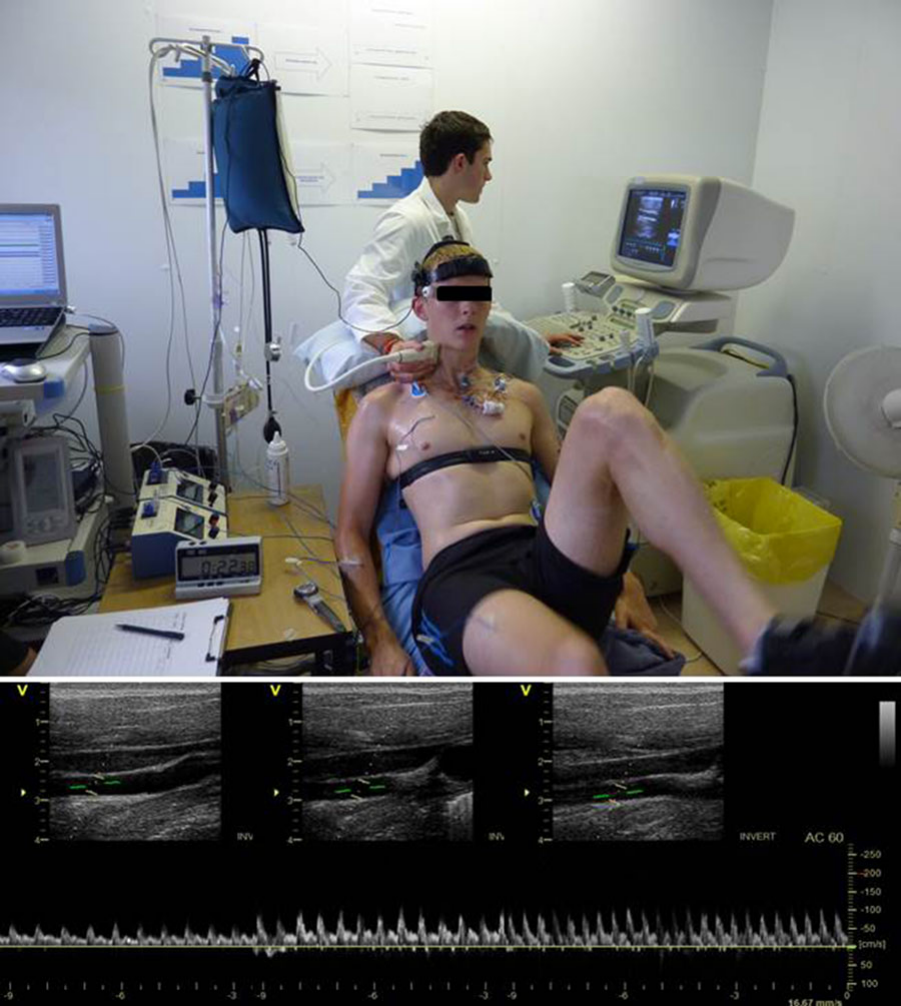
Experimental arrangement and ultrasound recording Photo depicting one of the participants in the study performing an incremental cycling test on a semi-recumbent cycle ergometer (Lode Angio, Groningen, the Netherlands) with a backrest inclination of 45 deg, while measurements of ICA and CCA blood flow were obtained at each stage. Representative images of real time ICA blood velocity recordings at rest, submaximal and peak exercise are shown.

The experimental days (visits 4 and 5) included three semi-recumbent incremental cycling exercise tests consisting of five 3 min stages of increasing intensities to WR_max_ (Figs 1 and 2). In the first experimental trial, incremental cycling was completed in the following conditions: (1) in a ‘control’, hydrated state; (2) ‘dehydrated’ (DEH), ∼5 min after 2 h of submaximal cycling without fluid ingestion; and (3) rehydrated (REH), after 1 h recovery with full fluid replacement. Work rates for control and REH were the same (67 ± 3, 134 ± 5, 202 ± 8, 269 ± 11 and 336 ± 14 W, corresponding to 20, 40, 60, 80 and 100% of WR_max_) but in anticipation of a reduced exercise capacity when dehydrated, WR in DEH was reduced by 20% to maintain the same number of exercise stages and test duration with work rates set at 54 ± 2, 108 ± 4, 161 ± 7, 215 ± 9 and 269 ± 11 W, respectively. In the second experimental trial (i.e. euhydration trial), carried out on a separate day, participants completed the same incremental and prolonged exercise protocols, but hydration was maintained through fluid ingestion according to the body mass loss. Fluid was provided in aliquots of ∼160 ml every 10 min during the 2 h of submaximal exercise and also pre- and post-incremental exercise at the same work rates. The euhydration trial was used to isolate the effect of dehydration on the observed haemodynamic responses to incremental exercise and to control for the effect of repeated exercise. In both trials, incremental exercise was performed in the heat (35°C, RH 50%) with pedal cadence maintained between 70 and 90 r.p.m. Participants were exposed to the environmental conditions for 1 h prior to commencement of the protocol.

In the dehydration trial, cerebral haemodynamics and blood samples from the brachial artery and left internal jugular vein were obtained simultaneously in the final minute of each exercise stage. Intestinal, skin and jugular venous temperatures and arterial and jugular venous pressures were recorded. The same measures were collected in the euhydration trial, except for the arterial–venous (a–v) blood sampling and jugular venous temperatures and pressures.

### Cerebral haemodynamics

Blood flow was obtained sequentially from the right CCA and internal carotid arteries (ICA) at rest and in the final minute of each work rate using an ultrasound system (Vivid 7 Dimension, GE Healthcare, UK) equipped with a 10 MHz linear array transducer. Measurements were performed by an experienced sonographer with care taken to maintain sampling site and vessel insonation angle. Participants were seated on the cycle ergometer and encouraged to maintain a consistent head position for optimal ultrasound scanning. ICA and CCA measurements were typically taken ∼1.0–1.5 cm above and ∼1.5 cm below the carotid bifurcation, respectively (Sato *et al*. 2011; Willie *et al*. 2012) with settings maintained across the protocol. Test–retest reliability was assessed during pilot studies and the coefficient of variation for CCA and ICA volume flow measurements at rest were 2.8 ± 0.9% and 4.3 ± 1.0%, and during exercise were 5.3 ± 1.6% and 5.0 ± 1.6%, respectively. For calculation of blood flow, two-dimensional brightness mode images for CCA and ICA diameter were taken, followed by pulse-wave measurements for the assessment of time-averaged mean velocity. Systolic and diastolic diameters were measured with the mean diameter calculated as systolic diameter × 1/3 + diastolic diameter × 2/3.

Time-averaged mean flow velocity (TAM *V*; cm s^−1^) was measured in pulse-wave mode, taken as the average of three continuous 12 s periods. Average diameter and flow velocity profiles were made from ≥15 cardiac cycles to attenuate respiration artefacts. The sample volume was maintained at the centre of the vessel lumen and adjusted to cover its width. Care was taken to ensure a consistent insonation angle below 60 deg. Mean flow velocity profiles were traced automatically and analysed offline for determination of TAM *V* (EchoPAC BT12, Version: 112, GE Healthcare, Norway). Blood flow (ml min^−1^) was then calculated by mean flow velocity × cross-sectional area (CSA: π × (mean diameter/2)^2^); blood flow = TAM *V* × CSA × 60.

Due to technical limitations, blood flow measurements were made in all work rates except the 100% stage in control and rehydration conditions. Blood flow in these stages was estimated using the individual decline in MCA *V*_mean_. MCA *V*_mean_ was measured using 2 MHz pulsed trans-cranial Doppler ultrasound (Doppler-Box, Compumedics DWL, Singen, Germany). The right MCA was insonated through the temporal ultrasound window at a depth of 45–60 mm. Signal quality was optimised according to Aaslid *et al*. (1982).

### Catheter placement and blood sampling

While resting with a slight head-down tilt; catheters for blood sampling, blood pressure (mean arterial pressure, MAP), internal jugular venous pressure and blood temperature were inserted into the brachial artery of the non-dominant arm and after local anaesthesia (2% lidocaine) in the left internal jugular vein (Double Lumen Catheter, 16 gauge, 2.3 mm; Multi-Med M2716HE, Edwards Lifesciences, USA) using the Seldinger technique, and advanced to the jugular bulb. For measurement of jugular venous blood temperature, a thermistor (T204-D, PhysiTemp, Clifton, NJ, USA) was inserted through the catheter and connected to a thermocouple meter (TC-2000, Sable Systems, NV, USA). The internal jugular catheter was inserted under ultrasound guidance and catheters were regularly flushed with 0.9% saline to maintain patency. The time from catheterisation to the commencement of resting measurements was ∼1 h.

### Blood variables

Arterial and jugular venous blood samples were drawn into pre-heparinised syringes and analysed immediately for blood gas variables (ABL 800 FLEX, Radiometer, Copenhagen, Denmark) corrected for blood temperature in the internal jugular vein. The analyser was calibrated at regular intervals in accordance with manufacturer guidelines. Additional arterial and jugular venous blood was collected in 2 ml syringes and transferred to EDTA tubes, centrifuged and separated. Plasma adrenaline and noradrenaline were subsequently determined using an enzyme immunoassay kit (DEE6500 2-CAT, Demeditec Diagnostics GmbH, Kiel, Germany). Blood samples were also collected directly in stop solution (Gorman *et al*. 2003; Kalsi & González-Alonso, 2012). Plasma ATP was then determined using the luciferin–luciferase technique by a luminometer with three automatic injectors (Orion Microplate Luminometer, Bethold Detection System GmbH, Pforzheim, Germany).

### Heart rate, blood pressure and temperatures

Heart rate was obtained from a chest strap (Polar Electro, Kempele, Finland). Arterial and internal jugular venous pressure waveforms were recorded using transducers (Pressure Monitoring Kit, TruWave, Edwards Lifesciences, Germany) zeroed at the level of the right atrium in the midaxillary line (arterial) and at the level of the tip of the catheter (jugular venous). Arterial pressure waveforms were sampled at 1000 Hz, amplified (BP amp, ADInstruments, Oxford, UK) and connected to a data acquisition unit (Powerlab 16/30, ADInstruments) for offline analysis. Intestinal temperature was measured using an ingestible telemetry pill (HQInc., Palmetto, FL, USA) and mean skin temperature from four sites (standard weightings of chest, abdomen, thigh and calf; Ramanathan, 1964) was obtained using a wired thermocouple system (TC-2000, Sable Systems, Las Vegas, NV, USA).

### Calculations

Cerebral vascular conductance (CVC) indices were calculated by dividing blood flow in the ICA and CCA, and MCA *V*_mean_, by cerebral perfusion pressure (difference between MAP and jugular venous pressure). Arterial oxygen content was used to quantify O_2_ delivery through the MCA and ICA, respectively. CMRO_2_ and CO_2_ production indices were calculated as 2 × ICA flow multiplied by the arterial–venous (a–v) O_2_ difference and/or venous–arterial (v–a) CO_2_ difference. Whole blood CO_2_ content was also calculated (Douglas *et al*. 1988).

### Data analysis

A one-way repeated-measures ANOVA was used for the assessment of changes over time (i.e. rest and increasing exercise intensities). Where significant differences were found, appropriate *post hoc* analysis were made using the Dunn–Sidak correction. Where applicable, measured variables between conditions were analysed using a two-way repeated-measures ANOVA in which condition (control, DEH and REH) and exercise phase (rest, 20, 40, 60, 80 and 100%) were the main factors. Multiple regression for within-subject repeated measures was used for the analysis of the relationship between blood flow and blood gas variables and temperatures (Bland & Altman, 1995; Slinker & Glantz, 2008). Statistical significance was set at *P* < 0.05 and all analyses were made using IBM SPSS Statistics (Version 20, IBM Corporation, Armonk, NY, USA).

## Results

### Hydration and temperature

In the dehydration trial (Fig. 1), body mass in DEH was lower compared to control (75.8 ± 2.7 *vs*. 78.2 ± 2.7 kg, corresponding to a 3.1 ± 0.3% body mass loss, *P* < 0.01), and was restored in REH (77.7 ± 2.9 kg). DEH was accompanied by an increased arterial and venous haemoglobin concentration ([Hb]) (*P* < 0.01; Table 1), indicative of a reduction in blood volume, whereas REH restored these responses. Prior to exercise, intestinal and internal jugular venous temperatures were higher in DEH compared to control (38.3 ± 0.1 *vs*. 36.8 ± 0.1 and 37.7 ± 0.1 *vs*. 36.5 ± 0.1°C, respectively, both *P* < 0.001; Fig. 6*C*), but were restored to control values in REH (36.5–36.8°C). In DEH, both intestinal and blood temperature remained elevated and increased with work rates to a peak of 38.2 ± 0.1°C (*P* < 0.01; Fig. 6*C*). In control, intestinal and internal jugular venous temperature increased progressively to 37.4 ± 0.1 and 37.9 ± 0.1°C, with similar responses observed during REH. Mean skin temperature (*T*_sk_) was unchanged across exercise intensities and between incremental conditions (33.8 ± 0.3, 32.6 ± 0.4 and 33.1 ± 0.3°C in control, DEH and REH, respectively). Heart rate followed the same pattern, with peak values being similar in all three conditions (179 ± 4, 184 ± 2 and 179 ± 3 beats min^−1^ in control, DEH and REH, respectively).

**Table 1:** Blood responses to incremental cycling exercise

			Incremental cycling exercise (%WR_max_ in Control)					
			
			Rest	20%	40%	60%	80%	100%
Hb (g l^−1^)	Control	a	141 ± 5	145 ± 4*	147 ± 4*	149 ± 4*	154 ± 4*	158 ± 4*
		v	140 ± 5	144 ± 4*	146 ± 4*	148 ± 4*	152 ± 4*	156 ± 4*
	Dehydration	a	152 ± 4	151 ± 4	152 ± 4	154 ± 4*	156 ± 4*	—
		v	152 ± 4	148 ± 3	149 ± 3	149 ± 4	152 ± 3†	—
	Rehydration	a	140 ± 3	141 ± 3	142 ± 3	146 ± 3*	149 ± 2*	148 ± 4*
		v	140 ± 4	139 ± 3	142 ± 3	147 ± 3*	147 ± 3*	148 ± 4*
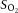 (%)	Control	a	98.5 ± 0.2	97.7 ± 0.1*	97.8 ± 0.2*	97.5 ± 0.3*	97.3 ± 0.4*	96.6 ± 0.4*
		v	64.7 ± 1.0	66.0 ± 1.5	68.7 ± 1.0*	67.0 ± 1.1	64.9 ± 1.5†	61.0 ± 2.1†
	Dehydration	a	98.1 ± 0.4	97.4 ± 0.1	97.4 ± 0.1	97.5 ± 0.4	97.9 ± 0.2	—
		v	65.7 ± 0.8	63.2 ± 1.2*	64.0 ± 0.9	63.9 ± 1.3	63.4 ± 2.0	—
	Rehydration	a	98.5 ± 0.1	97.2 ± 0.5*	97.4 ± 0.2*	97.2 ± 0.1*	97.2 ± 0.3*	97.0 ± 0.7*
		v	65.9 ± 1.4	65.0 ± 1.5	65.4 ± 2.2	65.7 ± 1.9	65.6 ± 3.2	65.9 ± 6.3
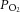 (mmHg)	Control	a	99 ± 3	90 ± 2*	94 ± 3	94 ± 4	96 ± 4	97 ± 4
		v	36 ± 1	36 ± 1	38 ± 1*	38 ± 1*	39 ± 1*	40 ± 1*
	Dehydration	a	101 ± 4	91 ± 2	90 ± 2	94 ± 4	96 ± 2	—
		v	40 ± 1	37 ± 1*	37 ± 1*	39 ± 2*	38 ± 1*	—
	Rehydration	a	105 ± 2	93 ± 3*	89 ± 2*	89 ± 2*	91 ± 3*	96 ± 8*
		v	37 ± 1	36 ± 1	36 ± 1	38 ± 1	38 ± 2	38 ± 2
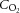 (ml l^−1^)	Control	a	192 ± 6	195 ± 6	199 ± 5*	201 ± 5*	206 ± 5*	211 ± 6*
		v	127 ± 4	131 ± 5	138 ± 5†	137 ± 4	136 ± 5	131 ± 5
	Dehydration	a	206 ± 5	203 ± 5	203 ± 5	207 ± 6	210 ± 5*	—
		v	140 ± 2	134 ± 6	131 ± 3	132 ± 3	133 ± 4	—
	Rehydration	a	191 ± 4	189 ± 4	191 ± 4	195 ± 4*	200 ± 3*	203 ± 5*
		v	127 ± 2	124 ± 2	127 ± 3	132 ± 3	132 ± 6	124 ± 4
pH	Control	a	7.39 ± 0.01	7.38 ± 0.01*	7.36 ± 0.01*	7.36 ± 0.01*	7.36 ± 0.01	7.31 ± 0.01*
		v	7.33 ± 0.01	7.32 ± 0.02	7.32 ± 0.01	7.32 ± 0.01	7.32 ± 0.01	7.26 ± 0.01*
	Dehydration	a	7.40 ± 0.01	7.38 ± 0.01	7.38 ± 0.01	7.38 ± 0.03	7.41 ± 0.02	—
		v	7.34 ± 0.01	7.32 ± 0.01	7.30 ± 0.03	7.33 ± 0.02	7.38 ± 0.01*	—
	Rehydration	a	7.38 ± 0.01	7.37 ± 0.01	7.37 ± 0.01	7.37 ± 0.01	7.37 ± 0.01	7.34 ± 0.03
		v	7.33 ± 0.01	7.32 ± 0.02	7.32 ± 0.01	7.32 ± 0.01	7.33 ± 0.01	7.32 ± 0.02

Values are means ± SEM for 10 subjects. Control, dehydration and rehydration incremental exercise tests are represented.

* Different from rest, *P* < 0.05.

† Different from previous intensity, *P* < 0.05. 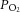, partial pressure of O_2_; 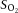, O_2_ saturation of the blood; 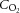, O_2_ content of the blood.

In the euhydration trial, body mass was the same at the start of each of the three incremental cycling tests. Prior to exercise, intestinal temperature was higher in the second and third test, compared to the first control test (37.8 ± 0.2 and 37.2 ± 0.1 *vs*. 37.0 ± 0.1°C; *P* < 0.05). During exercise, intestinal temperature increased with exercise intensity and reached 37.8 ± 0.1, 37.5 ± 0.1 and 37.4 ± 0.1°C, at exhaustion. Similarly to the dehydration trial, mean *T*_sk_ was unchanged across exercise intensities and between incremental tests (33.3 ± 0.2, 32.7 ± 0.3 and 33.3 ± 0.2°C, respectively). Heart rate was elevated prior to the second test compared to the first, but peak heart rate was not different (176 ± 2, 176 ± 3 and 177 ± 3 beats min^−1^, in the first, second and third tests, respectively).

### Brain haemodynamics and metabolism

**Figure 3:**
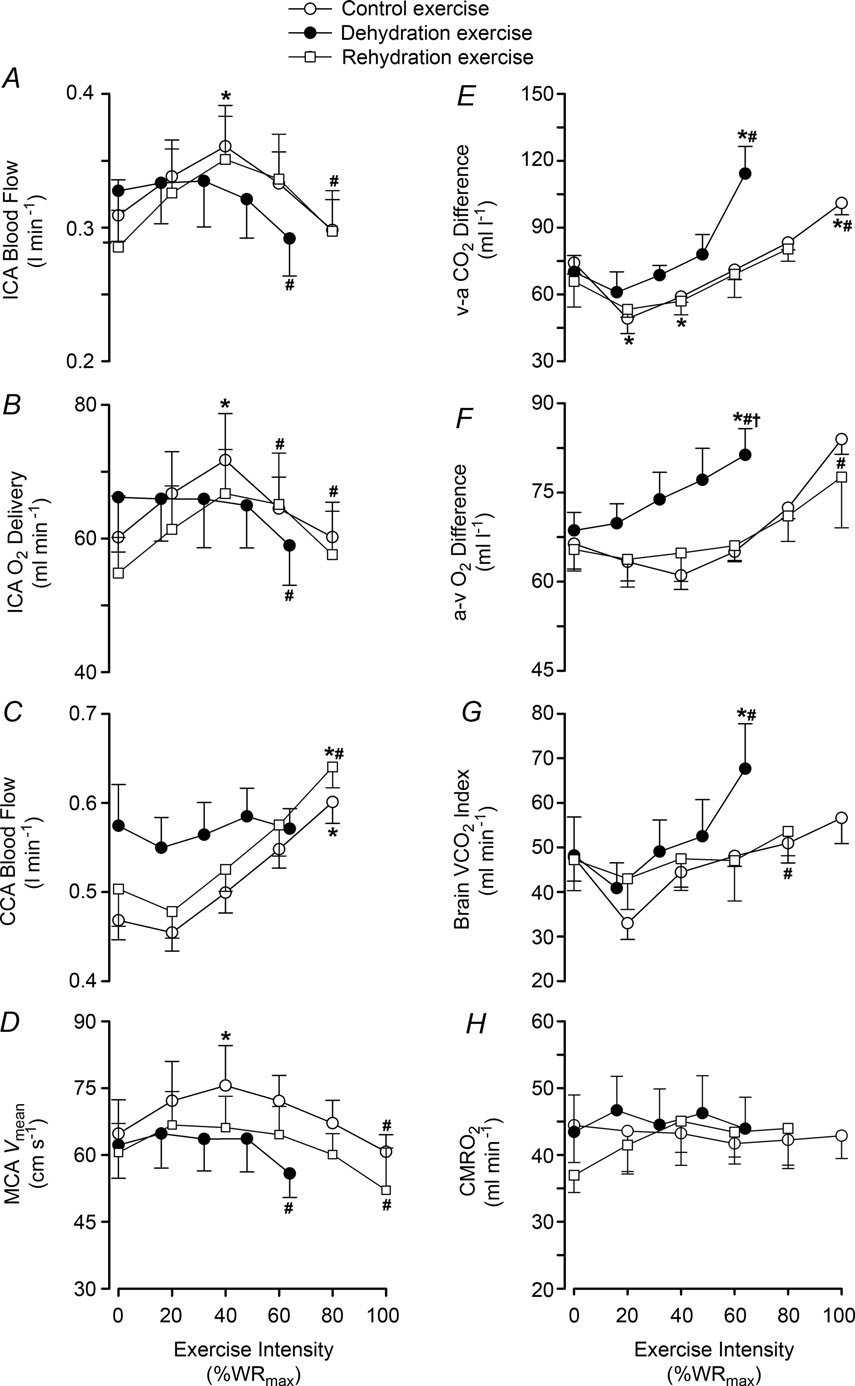
Cerebral haemodynamics and oxygen parameters during incremental exercise in different hydration states Left panel: internal carotid artery blood flow (*A*), ICA oxygen delivery (*B*), common carotid artery blood flow (*C*) and middle cerebral artery velocity (*D*). Right panel: jugular venous to arterial CO_2_ difference (v–a CO_2_; *E*), arterial to jugular venous oxygen difference (a–v O_2_; *F*), brain CO_2_ release (*G*), and brain oxygen uptake (CMRO_2_; *H*) for control (open circles), dehydration (filled circles) and rehydration (open squares) conditions. Values are mean ± SEM. *P* values represent ANOVA results. *P < 0.05 *vs*. rest, #*P* < 0.05 *vs*. sub-maximal exercise (i.e. ∼40% WR_max_).

In control in the dehydration trial, ICA blood flow and MCA *V*_mean_ increased by ∼17 ± 2% from rest to submaximal exercise and thereafter declined to resting values (both *P* < 0.05; Fig. 3*A* and *D*). Conversely, during DEH, ICA blood flow did not increase from rest to moderate exercise, but declined to below resting values at WR_max_ (−11% *vs*. rest, *P* < 0.05). ICA blood flow responses to REH were similar to control. In all conditions, the decline in blood flow at high exercise intensities was associated with reductions in vessel diameter and blood velocity. In contrast to ICA blood flow, CCA blood flow did not change during low intensity exercise in control, but increased progressively with further increases in exercise intensity (rest = 0.47 ± 0.02 *vs*. 0.60 ± 0.02 l min^−1^, *P* < 0.01) (Fig. 3*C*). During DEH, CCA blood flow was elevated (*P* < 0.05) at the start of exercise and did not change throughout incremental exercise. CCA blood flow responses to REH incremental exercise were similar to control. The increases in CCA blood flow in control and REH were associated with increases in blood velocity (*P* < 0.05). In the euhydration trial, ICA and CCA blood flow, and MCA *V*_mean_ were similar at rest and during incremental exercise.

**Figure 4:**
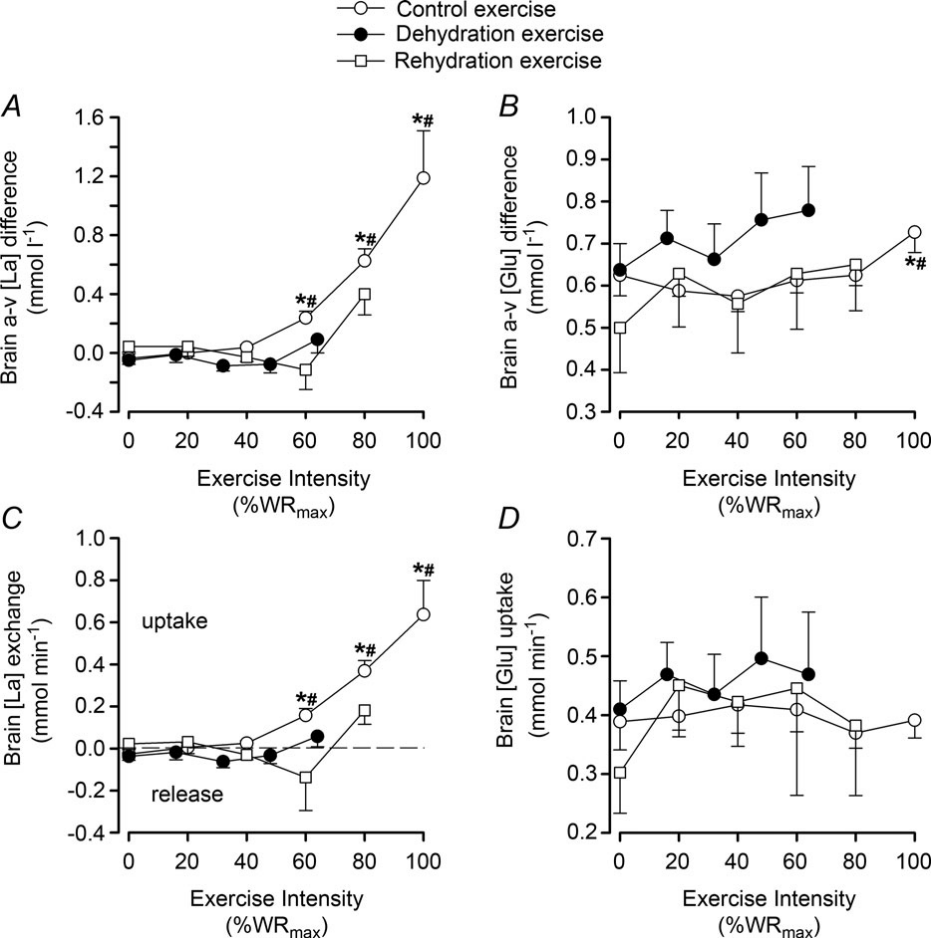
Brain lactate and glucose uptake during incremental exercise Brain a–v lactate [La] and glucose [Glu] concentration differences (*A* and *B*) and [La] and [Glu] uptake/exchange (*C* and *D*) during the 3 incremental tests. Exchange was calculated as the product of 2 × ICA blood flow and a–v difference. Data are means ± SEM for 7 subjects. *P < 0.05 *vs*. rest; #*P* < 0.05 *vs*. sub-maximal exercise.

At rest, ICA O_2_ delivery, a–v O_2_ and v–a CO_2_ difference, and CMRO_2_ and brain rate of CO_2_ production (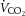) indices were not significantly different across the three experimental conditions of the dehydration trial. From rest to sub-maximal exercise (40% WR_max_) in control, ICA O_2_ delivery increased, v–a CO_2_ difference decreased, while the a–v O_2_ difference was unchanged (Fig. 3*B*, *E* and *F*). When exercise intensity became strenuous (≥60%), ICA O_2_ delivery declined to baseline values, as with ICA blood flow, and v–a CO_2_ and a–v O_2_ difference increased progressively to exhaustion (∼32% increase *vs*. rest, *P* < 0.05). Additionally, there was a progressive increase in brain 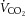 index up to WR_max_ (Fig. 3*G*). During DEH, ICA O_2_ delivery remained constant up to 60% WR_max_, before declining to below resting values_._ Moreover, v–a CO_2_ difference, a–v O_2_ difference and brain 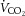 index were elevated at WR_max_ (*P* < 0.05). ICA O_2_ delivery was somewhat restored in REH whereas v–a CO_2_ and a–v O_2_ difference, and brain 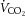 index were similar to control. Overall, these responses resulted in a maintained CMRO_2_ index at rest and throughout exercise to exhaustion (Fig. 3*H*). Brain a–v lactate concentration ([La]) difference was maintained at sub-maximal exercise intensities in control conditions before increasing at WR_max_, resulting in net uptake of [La] by the brain (Fig. 4*A* and *C*). Conversely, in DEH and REH, a–v [La] was unchanged. Brain a–v glucose concentration ([Glu]) difference was stable in all conditions (except WR_max_ in control conditions), resulting in a stable uptake of glucose across exercise intensities (Fig. 4*B* and *D*).

### Blood pressure and vascular conductance

**Figure 5:**
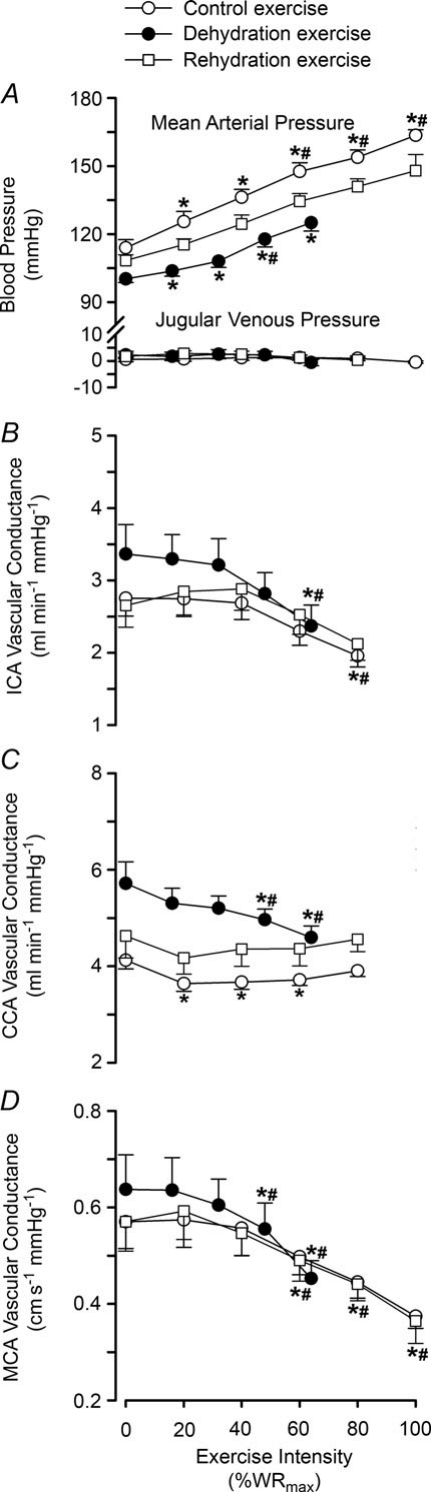
Cerebral vascular conductance and perfusion pressure during incremental exercise in different hydration states Mean arterial and jugular venous pressures (*A*), internal carotid, common carotid and middle cerebral artery vascular conductance indices (*B*–*D*) for control (open circles), dehydration (filled circles) and rehydration (open squares) conditions. Values are mean ± SEM. *P* values represent ANOVA results. *P < 0.05 *vs*. rest, #*P* < 0.05 *vs*. sub-maximal exercise (i.e. ∼40% WR_max_). Significance for control and rehydration were similar in panels *A*, *B* and *D*.

At rest and during incremental exercise in the dehydration trial, MAP was lower in DEH compared to control whereas jugular venous pressure was not different across incremental exercise conditions (*P* < 0.01; Fig. 5*A*). Brain perfusion pressure was therefore lower in DEH compared to control (*P* < 0.01). Concurrently, ICA, CCA and MCA vascular conductances were higher in DEH, compared to control and REH, at rest (*P* < 0.01; Fig. 5*B–D*). However, in all incremental exercise conditions, ICA and MCA vascular conductances were not different at sub-maximal exercise intensities before declining at WR_max_ (*P* < 0.05). During control, CCA vascular conductance declined from rest to sub-maximal exercise intensities before recovering to baseline values at WR_max_, whereas in DEH CCA vascular conductance continued to decline. In contrast to the haemodynamic alterations seen in the dehydration trial, in the euhydration trial MAP and ICA, CCA and MCA vascular conductance were similar at rest and throughout the three exercise tests.

### Cerebral blood flow, 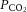, 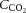 and temperature

**Figure 6:**
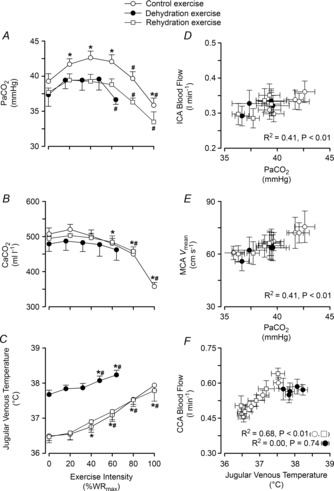
Relationships between cerebral perfusion and blood CO_2_ and temperature Left panel: 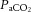 (*A*), arterial CO_2_ content (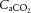, *B*), and jugular venous temperature responses to incremental exercise (*C*). Right panel: ICA blood flow and MCA *V*_mean_ group mean correlations with 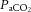 (*D* and *E*), and CCA blood flow group mean correlation to jugular venous temperature (*F*) in control (open circles), dehydration (filled circles) and rehydration (open squares). *P < 0.05 *vs*. rest, #*P* < 0.05 *vs*. sub-maximal exercise (i.e. ∼40% WR_max_). Unless presented, significance for control and rehydration were similar (i.e. panels *B* and *C*).

At rest, 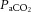 was not different across conditions. The transition from rest to exercise resulted in an increase in 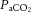 in all incremental exercise conditions that continued up to 40% WR_max_ in control, whereas in DEH and REH 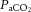 was unchanged above 20% WR_max_. Beyond sub-maximal intensities 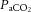 rapidly declined, by 6–7 mmHg, to below resting values in control (and REH), and by 3 mmHg in DEH (*P* < 0.05; Fig. 6*A*). Venous CO_2_ tension (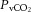) increased from rest to 60% WR_max_ in control conditions before declining to baseline values at WR_max_, whereas in DEH and REH 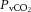 was unchanged throughout exercise (Table 2). At rest arterial CO_2_ content (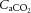) was lower in DEH compared to control and REH (479 ± 22 *vs*. 507 ± 17 and 495 ± 6 ml l^−1^; Fig. 6*B*). From rest to WR_max_, 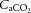 declined to below resting values in control (and REH; *P* < 0.05), but a similar decline was not apparent in DEH. Jugular venous CO_2_ content (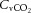) declined from rest to WR_max_ (581 ± 15 to 463 ± 11 ml l^−1^; *P* < 0.05) in control conditions, whereas in DEH and REH 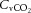 was unchanged throughout exercise (∼553 ± 4 ml l^−1^).

**Table 2:** Blood gases and metabolite responses to incremental exercise in different hydration states

			Incremental cycling exercise (% WR_max_ in Control)					
			
			Rest	20%	40%	60%	80%	100%
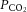 (mmHg)	Control	a	39 ± 1	42 ± 1*	43 ± 1*	42 ± 1*	40 ± 1*	36 ± 1*^,^†
		v	50 ± 1	52 ± 1	52 ± 1	53 ± 1*	52 ± 1	50 ± 1†
	Dehydration	a	37 ± 2	39 ± 1	39 ± 1	40 ± 2	37 ± 1†	—
		v	49 ± 1	50 ± 1	48 ± 2	47 ± 3	48 ± 2	—
	Rehydration	a	38 ± 1	39 ± 1	39 ± 1	39 ± 1	36 ± 1†	33 ± 1†
		v	49 ± 1	49 ± 1	49 ± 1	50 ± 1*	48 ± 2†	45 ± 4†
[HCO_3_^−^] (mmol l^−1^)	Control	a	23.5 ± 0.7	24.0 ± 0.6	23.1 ± 0.7†	23.0 ± 0.6	22.2 ± 0.6	18.7 ± 0.8
		v	23.6 ± 0.8	23.1 ± 1.1	23.6 ± 0.6	23.6 ± 0.7	23.2 ± 0.8	19.3 ± 0.5
	Dehydration	a	23.9 ± 0.7	23.2 ± 1.0	23.0 ± 0.9	23.1 ± 1.3	23.7 ± 1.1	—
		v	23.6 ± 0.8	23.1 ± 1.0	21.6 ± 1.6	23.4 ± 1.6	26.5 ± 0.6	—
	Rehydration	a	22.5 ± 0.6	22.4 ± 0.6	22.4 ± 0.6	22.2 ± 0.7	21.6 ± 0.7	19.2 ± 0.9
		v	23.1 ± 0.6	22.7 ± 0.6	22.7 ± 0.7	23.0 ± 0.7	20.5 ± 1.9	21.2 ± 0.4
ABE (mmol l^−1^)	Control	a	−1.1 ± 0.9	−0.3 ± 0.7	−1.3 ± 0.8†	−1.5 ± 0.8	−2.7 ± 0.7	−7.4 ± 1.0
		v	0.7 ± 1.0	0.3 ± 1.2	1.0 ± 0.7	1.0 ± 0.9	0.4 ± 1.0	−4.3 ± 0.5
	Dehydration	a	−1.7 ± 0.9	−1.7 ± 1.3	−1.9 ± 1.2	−2.0 ± 1.7	−1.4 ± 1.3	—
		v	0.6 ± 0.9	0.0 ± 1.2	−2.0 ± 2.1	−1.1 ± 2.2	2.7 ± 1.4	—
	Rehydration	a	−2.4 ± 0.7	−2.3 ± 0.8	−2.4 ± 0.8	−2.8 ± 0.9	−3.7 ± 0.9	−6.9 ± 1.0
		v	0.1 ± 0.8	−0.4 ± 0.8	−0.3 ± 0.8	0.0 ± 0.8	−3.4 ± 2.4	−2.6 ± 0.8
Lactate (mmol l^−1^)	Control	a	0.8 ± 0.1	1.3 ± 0.1*^,^†	1.7 ± 0.1*^,^†	2.8 ± 0.2*^,^†	5.6 ± 0.4*^,^†	11.3 ± 0.7*^,^†
		v	0.9 ± 0.1	1.3 ± 0.1*^,^†	1.6 ± 0.1*^,^†	2.6 ± 0.2*^,^†	5.0 ± 0.4*^,^†	10.1 ± 0.6*^,^†
	Dehydration	a	2.1 ± 0.2	1.9 ± 0.2*^,^†	1.6 ± 0.2*^,^†	1.7 ± 0.2*	2.6 ± 0.2*^,^†	—
		v	2.2 ± 0.2	1.9 ± 0.2*^,^†	1.7 ± 0.2*^,^†	1.7 ± 0.2*^,^†	2.4 ± 0.2†	—
	Rehydration	a	3.3 ± 0.3	2.9 ± 0.2*^,^†	2.5 ± 0.2*^,^†	2.8 ± 0.2	4.8 ± 0.2*^,^†	8.8 ± 0.3*^,^†
		v	3.3 ± 0.3	2.9 ± 0.2*^,^†	2.5 ± 0.2*^,^†	2.9 ± 0.2†	4.3 ± 0.2*^,^†	8.2 ± 0.3*^,^†
Glucose (mmol l^−1^)	Control	a	6.0 ± 0.2	6.0 ± 0.2	6.0 ± 0.2	5.9 ± 0.2	5.8 ± 0.2†	5.7 ± 0.2
		v	5.4 ± 0.2	5.4 ± 0.2	5.4 ± 0.2	5.3 ± 0.2†	5.2 ± 0.2†	5.0 ± 0.2†
	Dehydration	a	6.0 ± 0.2	5.6 ± 0.3*^,^†	5.2 ± 0.3*^,^†	5.0 ± 0.3*^,^†	4.7 ± 0.2*^,^†	—
		v	5.4 ± 0.2	4.9 ± 0.2*^,^†	4.6 ± 0.2*^,^†	4.2 ± 0.3*^,^†	4.0 ± 0.3*^,^†	—
	Rehydration	a	12.0 ± 0.7	11.2 ± 0.8*^,^†	10.6 ± 0.8*^,^†	9.7 ± 0.7*^,^†	8.3 ± 0.7*^,^†	6.6 ± 0.9*^,^†
		v	11.0 ± 0.5	10.0 ± 0.5*^,^†	9.4 ± 0.5*^,^†	8.6 ± 0.5*^,^†	7.4 ± 0.5*^,^†	6.2 ± 0.5*^,^†

Values are mean ± SEM for 10 participants. 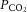, partial pressure of CO_2_; [HCO_3_^−^], sodium bicarbonate; ABE, acid–base excess; lactate and glucose for arterial (a) and internal jugular venous (v) blood. Rehydration values at 100% are *n* = 5.

* Different from rest, *P* < 0.05.

† Different from previous intensity, *P* < 0.05.

### Relationships between cerebral blood flow and 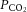, 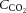, pH and temperature

At rest and throughout incremental exercise in all conditions, ICA blood flow (*R*^2^ = 0.41: Fig. 6*D*) and MCA *V*_mean_ (Coefficient of determination, *R*^2^ = 0.42: Fig. 6*E*) were correlated to changes in 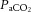 (both *P* < 0.01). In contrast, only non-significant correlations were observed for 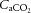 (*R*^2^ = 0.16), 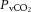 (*R*^2^ = 0.15) and 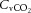 (*R*^2^ = 0.19; *P* = 0.15–0.85). Also, CCA (*R*^2^ = 0.05) and ICA (*R*^2^ = 0.13) blood flow, in all conditions, were not correlated to jugular venous pH (both *P* > 0.05). Lastly, CCA blood flow in control and REH was correlated to changes in jugular venous temperature (*R*^2^ = 0.68; *P* < 0.001: Fig. 6*F*), but not in DEH (*R*^2^ = 0.00; *P* = 0.74).

### Plasma catecholamines and ATP

**Figure 7:**
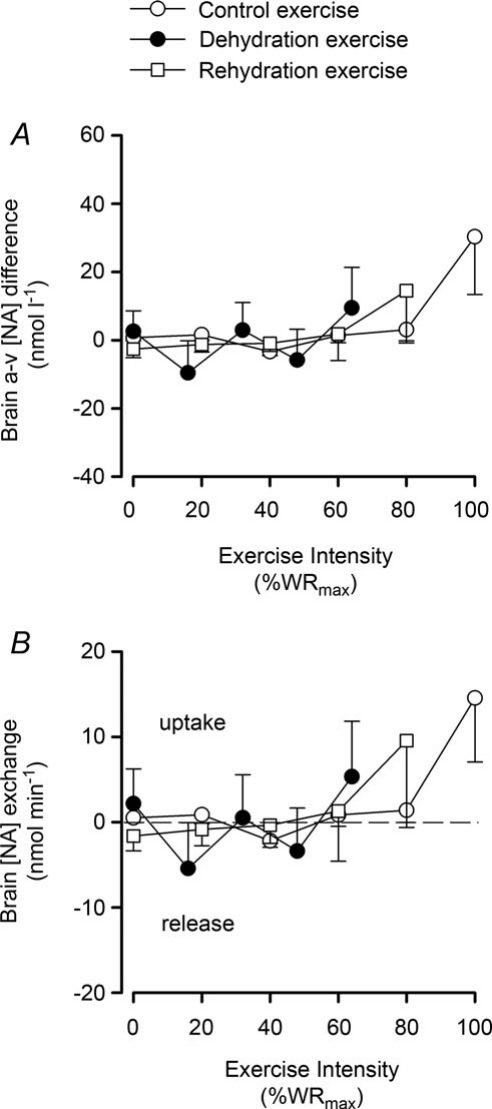
Brain noradrenaline (NA) exchange during incremental exercise Brain a–v noradrenaline concentration [NA] difference (*A*) and exchange (*B*) across the brain. Exchange was calculated as the product of 2 × ICA blood flow and a–v difference. Values are means ± SEM for 7 subjects.

**Figure 8:**
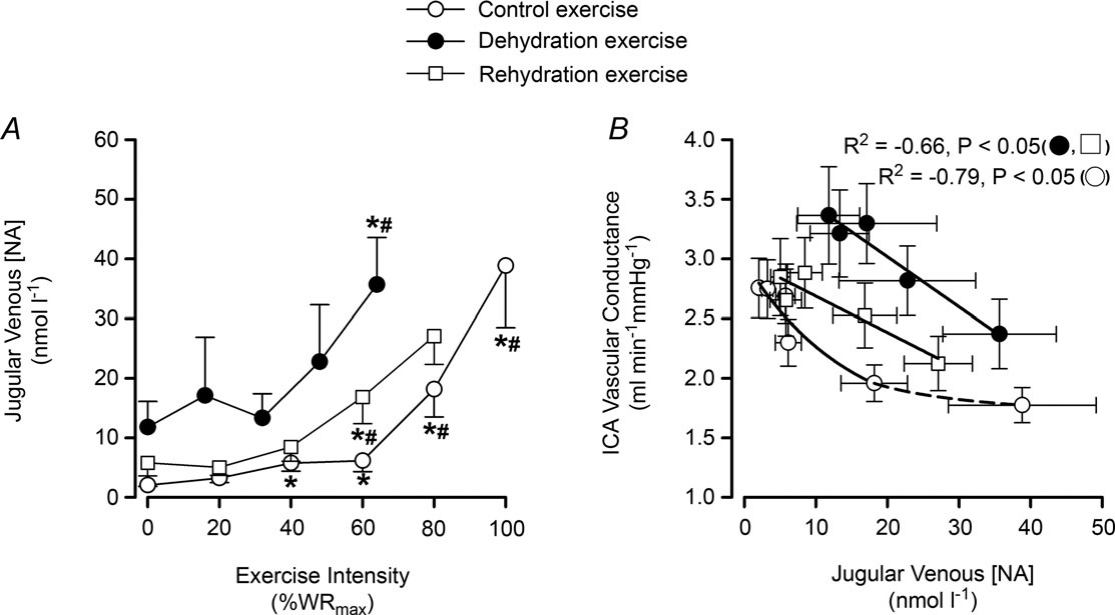
Jugular venous [NA] during incremental exercise and relationship of ICA vascular conductance and jugular venous [NA] Jugular venous [NA] and the relationship between ICA vascular conductance and jugular venous [NA] in control (open circles), dehydration (filled circles) and rehydration (open squares). *P < 0.05 *vs*. rest, #*P* < 0.05 *vs*. sub-maximal exercise (i.e. ∼40% WR_max_). Unless presented, significance for control and rehydration were similar.

At rest in DEH, arterial and jugular venous noradrenaline concentration ([NA]) was higher than control and rehydration (13 ± 4 *vs*. 3 ± 1 and 3 ± 1 nmol l^−1^ and 12 ± 4 *vs*. 2 ± 0.2 and 6 ± 2 nmol l^−1^, respectively; *P* < 0.05). From rest to WR_max_, arterial and jugular venous [NA] increased exponentially in all conditions to a peak of 43 ± 10, 69 ± 19 and 82 ± 21 nmol l^−1^, and 36 ± 8, 39 ± 10 and 27 ± 5 nmol l^−1^ in dehydration, control and rehydration, respectively. The a–v [NA] differences and exchange across the brain remained stable in the three trials (Fig. 7). The reductions in ICA vascular conductance were correlated to an increased jugular venous [NA] (control *R*^2^ = −0.79, dehydration and rehydration *R*^2^ = −0.66; *P* < 0.05: Fig. 8*B*). On the other hand, arterial and jugular venous adrenaline concentration ([A]) was not different among conditions at rest (1.1 ± 0.3 *vs*. 0.8 ± 0.2 and 0.8 ± 0.2 nmol l^−1^ and 1.0 ± 0.3 *vs*. 0.7 ± 0.1 and 0.6 ± 0.1 nmol l^−1^, respectively). Yet, from rest to WR_max_ in dehydration, control and rehydration conditions, [A] increased to a peak of 5.5 ± 1.9, 9.1 ± 2.2 and 7.7 ± 2.8 nmol l^−1^ in arterial and 6.5 ± 2.4, 8.5 ± 3.6 and 3.3 ± 1.1 nmol l^−1^ in venous plasma, respectively (all *P* < 0.05). Lastly, arterial plasma [ATP] increased in a curvilinear manner from similar values at rest (1058 ± 177 *vs*. 938 ± 128 and 1027 ± 199 nmol l^−1^) to WR_max_, and was higher in dehydration compared to control and rehydration at maximal intensities (1641 ± 189 *vs*. 1403 ± 221 and 1274 ± 188 nmol l^−1^; *P* < 0.05).

## Discussion

The novel findings of the present study were threefold. Firstly, during exercise in control conditions cerebral perfusion increased from rest to moderate exercise in the heat, before declining to baseline values prior to exhaustion. Secondly, dehydration accelerated the declines in blood flow and O_2_ delivery to the brain during incremental cycling exercise to exhaustion in association with a blunted perfusion pressure, reductions in 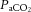 and increases in internal jugular venous [NA]. In contrast to the evident cerebral circulatory strain during the intense exercise stages, common carotid artery blood flow increased from rest to peak exercise in the control and rehydration conditions and remained unchanged with dehydration, indicating that the increase in blood flow to extra-cranial tissues was related to the increase in temperature (jugular blood). Finally, compensatory increases in brain O_2_ extraction maintained CMRO_2_ throughout exercise in association with a stable or increasing CO_2_ production. Collectively these findings suggest that the circulatory strain on the human brain during maximal exercise in the heat, even with dehydration, does not compromise CMRO_2_.

### Hydration and perfusion of the head

The current study demonstrates that CBF, blood velocity and O_2_ delivery are attenuated prior to the attainment of maximal work rate and that dehydration accelerates this restriction in cerebral perfusion. The decline in cerebral perfusion is in agreement with investigations in humans during graded incremental exercise (Moraine *et al*. 1993; Hellström *et al*. 1996; Sato *et al*. 2011) and intense constant load exercise, with and without heat stress (Nybo & Nielsen, 2001b, 2002; González-Alonso *et al*. 2004). We have extended these findings by obtaining direct measurements of anterior CBF under conditions that challenge the cardiovascular system to its capacity and examined the functional consequences of a diminished flow on CMRO_2_ during strenuous exercise.

The common carotid artery forms a major part of the extra-cranial circulation through to the ECA. During all incremental exercise conditions extra-cranial perfusion (CCA and calculated ECA flow: CCA – ICA) increased or was maintained. Strikingly, at rest prior to the dehydration test CCA blood flow was elevated by 25% whereas ICA blood flow was only modestly increased (∼6%), indicating a substantially augmented ECA blood flow compared to control when participants’ jugular venous and core temperatures were elevated by 1.2–1.5°C. Additionally, ECA blood flow increased by ∼50% from baseline to 80% WR_max_ (217 ± 30 to 307 ± 22 ml min^−1^) and achieved a similar peak value across interventions. These findings are consistent with an elevated extra-cranial blood flow with graded exercise in normothermic conditions (Hellström *et al*. 1996; Sato *et al*. 2011) and with passive heating at rest (Fan *et al*. 2008; Ogoh *et al*. 2013). Heat stress, with and without concomitant dehydration, results in a distinct cardiovascular strain (Sawka *et al*. 1979; Montain & Coyle, 1992a,b; González-Alonso *et al*. 1997; González-Alonso, 1998) and promotes redistribution of blood flow to the skin vascular beds for thermoregulatory purposes (Crandall *et al*. 2008; Crandall & González-Alonso, 2010; Johnson & Kellogg, 2010). Given that the ECA supplies the majority of the cutaneous circulation of the face and neck, an elevated blood flow to these regions is important for local convective heat exchange. Collectively these findings show contrasting blood flow adjustments across the different vascular beds of the head during strenuous exercise in the heat with both dehydration and euhydration.

### Mechanisms of cerebral and extra-cranial blood flow control

In all incremental exercise conditions attenuation in cerebral perfusion was coupled to a decline in cerebral vascular conductance, indicative of vasoconstriction and thus diminished vessel diameter (Fig. 5*B* and *D*). Alterations in 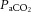 and blood CO_2_ content increased sympathetic nerve activity and concurrent changes in the intra- and extravascular milieu of vasoconstrictor and vasodilator signals may all play a role in restricting CBF (Paulson *et al*. 1990; Ide & Secher, 2000; Secher *et al*. 2008; Ogoh & Ainslie, 2009b). During strenuous exercise cerebral perfusion was associated with the decrease in 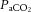 (Fig. 6*A*, *D* and *E*). Given that free CO_2_ accounts for only a minor portion of the CO_2_ in blood, we reasoned that 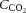 would indicate whether plasma and/or blood CO_2_ is important for the decline in cerebral perfusion. In contrast to the prominent association with 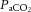, the correlation with arterial or jugular venous blood 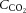 was non-significant, indicating that the cerebral circulation is sensitive to changes in free blood 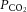 rather than to changes in CO_2_ bound to haemoglobin or buffered as bicarbonate in the arterial or venous vasculature. There is also controversy in regards to the role of cerebral venous *versus* arterial 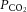 in regulation of brain blood flow (Peebles *et al*. 2007). The current study shows that the relationship between brain flow and 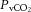 was not significant because of the maintenance or minimal changes in jugular 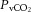. Furthermore, the impact of arterial 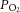 and HbO_2_ saturation on CBF is negligible in the present conditions because the changes in these variables during incremental exercise were too small to activate the oxygen-sensitive pathways of local CBF control (Willie *et al*. 2012). CO_2_ readily crosses the blood–brain barrier, altering the extracellular pH, and there is compelling evidence to suggest that pH has an independent effect on cerebral vessel vasoconstriction (Kontos *et al*. 1977a,*b*). However, there was no relationship between blood flow to the brain and jugular venous pH. Jugular venous pH may or may not reflect the environment of the extracellular space of the cerebral vasculature and the results suggest that pH is well maintained across the brain. The balance of pH (through the direct effects of CO_2_ and the buffering capacity of blood) is therefore important for the CBF response (Willie *et al*. 2014). Together, these findings point to a predominant influence of the arterial over that of the venous and thereby tissue CO_2_ in the regulation of CBF.

The present observations are consistent with the concept that the cerebral vasculature is highly sensitive to alterations in 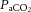 (Jørgensen *et al*. 1992; Secher *et al*. 2008), as evidenced by the ∼4% change in global and regional CBF per mmHg change in 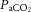 (expressed as the ‘cerebral CO_2_ reactivity’) (Madsen *et al*. 1993; Linkis *et al*. 1995; Willie *et al*. 2012), similar to that observed for regional CBF in the present study. The decline in 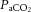 beyond moderate exercise intensities occurs in combination with the exponential increase in ventilation, which is accelerated under conditions that induce whole-body hyperthermia (Nybo & Nielsen, 2001b; Nybo *et al*. 2002; Wilson *et al*. 2006; Brothers *et al*. 2009a,b; Nelson *et al*. 2011; Ross *et al*. 2012). An important question is whether changing 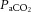 levels independently, or in combination with other related vasoconstrictor signals, are restricting CBF during intense exercise. We found that the decline in cerebral vascular conductance was associated with the large increase in jugular venous [NA]. An increase in circulating [NA] may influence cerebrovascular tone (Lee *et al*. 1976; Mitchell *et al*. 2009; Ogoh & Ainslie, 2009a; Seifert & Secher, 2011) and is associated with enhanced CMRO_2_ (King *et al*. 1952; Nemoto *et al*. 1996); however, controversy remains regarding its role within the cerebral vasculature (Strandgaard & Sigurdsson, 2008; van Lieshout & Secher, 2008). Irrespective of hydration status it appears that increasing jugular venous [NA] during intense exercise reflects increased local sympathetic vasoconstrictor activity and may explain some of the decline in CBF. However, increased circulating [NA] may not directly result in local vasoconstriction and the importance of sympathetic activity above and beyond the role of 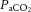 remains unclear.

In contrast to the close coupling between reductions in 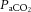 and cerebral perfusion, the relationship does not hold for the extra-cranial circulation (Sato *et al*. 2012; Ogoh *et al*. 2013), similar to that of peripheral vessels (Ainslie *et al*. 2005; Sato *et al*. 2012). The contrasting responses between the two vascular beds during exercise are interpreted to mean that blood flow is redistributed from the cerebral to the extra-cranial circulation (Sato *et al*. 2011). However, this is an unlikely scenario as preventing the decline in cerebral perfusion during passive hyperthermia through the clamping of end-tidal CO_2_ does not alter extra-cranial blood flow (Bain *et al*. 2013). Equally, reducing extra-cranial perfusion, through face cooling, appears to not influence MCA *V*_mean_ at rest or during light exercise (Miyazawa *et al*. 2012). Whilst 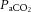 may not play an important role in the regulation of blood flow to the extra-cranial circulation, mechanisms involving temperature-sensitive pathways seem to do so. We observed for the first time a strong correlation between increases in common carotid artery blood flow and internal jugular venous temperature during control and REH incremental exercise (Fig. 6*F*). Additionally, with a rising blood temperature during incremental exercise in all three exercise conditions (up to 1.1°C), the plasma concentration of the potent intravascular vasodilator ATP increased in arterial blood; a potential mechanism for the temperature-related increase in regional perfusion (Pearson *et al*. 2011; González-Alonso, 2012; Kalsi & González-Alonso, 2012). Irrespective of the mechanisms, the progressive increase in extra-cranial perfusion may be an important pathway by which heat is locally dissipated to regulate temperature of the tissues within the head (Sato *et al*. 2011). Collectively, these data suggest that cerebral perfusion is restricted with a declining cerebral vascular conductance via a net increase in vasoconstrictor activity. Alterations in 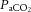 are the primary mechanism for regulation of cerebrovascular tone, but not extra-cranial vessel conductance.

### Is brain oxygen consumption compromised with dehydration during maximal incremental exercise?

An important question is whether central nervous system activity, and thus cerebral metabolic demand, rise sufficiently during strenuous exercise to increase CMRO_2_ and whether reductions in flow result in a compromised CMRO_2_. A major finding of the present study was that CMRO_2_ was not compromised throughout incremental exercise across exercise conditions in spite of an attenuated perfusion at maximal intensities. This response was met by an increased O_2_ extraction during maximal exercise, a response enhanced with dehydration. Our findings of an enhanced O_2_ extraction and a maintained CMRO_2_ are similar to observations during constant load sub-maximal (Ide & Secher, 2000; Nybo *et al*. 2002; González-Alonso *et al*. 2004; Secher *et al*. 2008) and maximal exercise (Scheinberg *et al*. 1954; González-Alonso *et al*. 2004). Nevertheless, the possibility exists that CMRO_2_ is somewhat suppressed during maximal exercise and dehydration due to reduced O_2_ supply. In this light, strenuous exercise with hyperthermia increases CMRO_2_, a response attributed to the requirement of an increased neuronal activity associated with mental effort and the *Q*_10_ effect of temperature on brain metabolism (Nybo *et al*. 2002). A marked reduction in O_2_ supply might lower intracellular 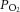 to an extent that affects metabolic fluxes and challenges cerebral metabolism and motor function (Gjedde *et al*. 2005; Nybo & Rasmussen, 2007; Rasmussen *et al*. 2007, 2010; Seifert *et al*. 2009). However, in spite of the 20% reductions in perfusion observed across conditions from submaximal to maximal exercise, it is unlikely that the capillary to intracellular 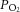 gradient was reduced to the extent that would compromise CMRO_2_ given that fractional oxygen extraction increased from 34% at rest to 39% at maximal exercise and was thereby within the range of adequate cerebral tissue oxygenation (Gjedde *et al*. 2005). This notion is consistent with the parallel observations that brain glucose uptake was well-maintained across exercise intensities and hydration conditions and lactate uptake was maintained or elevated (Fig. 4). Whilst it is difficult to speculate on the alterations within the deep structures of the brain, the current data suggest that brain oxygen consumption is not reduced during intense exercise in the heat, with and without concomitant dehydration.

### Methodological considerations

There are several methodological considerations in the present study. Firstly, blood flow measurements were made in the right CCA and ICA, whereas the vessels on the left-hand side of the anterior circulation and the vessels of posterior circulation were not measured. In regard to the anterior circulation, side-to-side blood flows at rest and during exercise are similar (Schöning *et al*. 1994; Sato *et al*. 2011; Willie *et al*. 2012). Secondly, blood flow measurements were made by one sonographer. Upon the transition from CCA to ICA ultrasound scans, a temporal lag and minor shift in sample area may occur. Care was taken to ensure a consistent measuring site for each participant and the use of duplex ultrasound allowed the continued monitoring of sample position. Thirdly, in contrast to previous literature observing the right internal jugular vein, we obtained venous blood samples from the left internal jugular vein. Asymmetry may exist in the venous drainage of the brain with the often larger right internal jugular vein draining the hemispheres and the left internal jugular vein draining the subcortical areas (Seifert & Secher, 2011). However, similar resting values for blood parameters and a–v O_2_ difference values are reported in the two jugular veins (Gibbs *et al*. 1942; Munck & Lassen, 1957). Moreover, comparable a–v O_2_ difference dynamics is observed during incremental exercise based on right jugular vein blood samples (Ide *et al*. 1999). We therefore assumed equal blood flow and O_2_ extraction in the left and right sides of the brain to estimate the CMRO_2_ index. Thirdly, the CMRO_2_ index underestimates the global CMRO_2_ because blood flow through the posterior circulation is not considered. The posterior portion of the brain is supplied by the two vertebral arteries (VAs) that anastomose to form the basilar artery before joining the circle of Willis, and their contribution to total brain blood flow is ∼20% at rest (Zauner *et al*. 1997). VA flow increases progressively with graded exercise intensities, in contrast to the anterior circulation (ICA) (González-Alonso *et al*. 2004; Sato *et al*. 2011, 2012). Thus, if we assume that VA blood flow increases, or follows the same pattern as the ICA, CMRO_2_ would remain unchanged during exercise in the conditions of the present study. Finally, we were unable to obtain satisfactory ultrasound images during the final stage (100%) in control and rehydration conditions. Blood flow in these stages, used for the calculation of CMRO_2_, was estimated using the percentage decline in MCA *V*_mean_ from the 80 to 100% work rate. This assumption has been used to assess changes in flow and CMRO_2_ during maximal exercise (Fisher *et al*. 2013).

### Conclusion

The present findings demonstrate that dehydration restricts CBF during strenuous exercise. The blunted CBF was associated with a decline in vascular conductance and 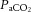 and an increase in systemic and jugular venous noradrenaline, indications of an enhanced vasoconstrictor activity. Cerebral oxygen extraction was increased during strenuous exercise, more so when perfusion was challenged with dehydration. In contrast, extra-cranial perfusion increased, mirrored by increases in blood temperature. Thus, reductions in cerebral perfusion and cerebral vascular conductance during maximal exercise in different hydration states does not appear to negatively impact CMRO_2_ because of compensatory increases in cerebral oxygen extraction.

## Abbreviations

a: arterial
[A]: plasma adrenaline concentration
CBF: cerebral blood flow
CCA: common carotid artery
carbon dioxide content in the blood
CMRO_2_: cerebral metabolic rate for oxygen
DEH: dehydrated
ECA: external carotid artery
ICA: internal carotid artery
MAP: mean arterial pressure
MCA *V*_mean_: middle cerebral artery mean blood velocity
[NA]: plasma noradrenaline concentration
partial pressure of arterial carbon dioxide
partial pressure of venous carbon dioxide
REH: rehydrated
v: venous
rate of CO_2_ production
peak aerobic capacity
WR_max_: maximal work rate
